# Characteristics and correlation of body fat distribution and brachial-ankle pulse wave velocity in adults aged 20–59 years: a cross-sectional study

**DOI:** 10.1186/s12872-023-03597-x

**Published:** 2024-01-02

**Authors:** Shengya Wang, Haiyan Shi, Laiyuan Luo, Hui He

**Affiliations:** 1 Department of Exercise Physiology, Beijing Sport University, Beijing, 100084 China; 2 China Institute of Sport and Health Science, Beijing Sport University, Beijing, 100084 China

**Keywords:** Fat distribution, Brachial-ankle pulse wave velocity, Anthropometric measures, Vascular stiffness

## Abstract

**Background:**

Fat distribution is closely related to vascular stiffness. This study aimed to investigate age and sex differences in fat distribution and brachial-ankle pulse wave velocity (baPWV), and the association between fat parameters and baPWV.

**Methods:**

A total of 10,811 participants aged 20–59 years were recruited. Measures included waist and hip circumference, waist-to-hip ratio (WHR), body mass index (BMI), percentage body fat (PBF), subcutaneous fat area (SFA), visceral fat area (VFA), and baPWV.

**Results:**

The results confirm that fat accumulates with age and that men tend to carry more abdominal fat than women in the same age group. The findings also indicate that baPWV increases with age and is significantly higher in men than in women in the same age group. In addition, WHR, VFA, and baPWV were more strongly correlated than baPWV and BMI, SFA, and PBF. Finally, the effects of age, PBF, WHR, and VFA on baPWV were greater for the higher quantiles.

**Conclusions:**

There are age and sex differences in fat distribution and baPWV. Abdominal obesity is more closely linked to arterial stiffness than overall obesity, and people with higher baPWV are more affected by obesity parameters.

**Supplementary Information:**

The online version contains supplementary material available at 10.1186/s12872-023-03597-x.

## Background

The prevalence of obesity has doubled since 1980 in more than 70 countries and continues to rise in most other countries [1]. Obesity is closely linked to a range of diseases that include neurological, digestive, respiratory, musculoskeletal, and infectious disorders, especially cardiovascular diseases (CVD) [1, 2]. In 2015, almost 70% of high body mass index (BMI) deaths were caused by CVD, and more than 60% of those deaths occurred in obese individuals [1]. China has the highest number of overweight and obese people globally [3]; CVD is the leading cause of death there [4], and CVD and obesity are now considered severe public health problems.

Obesity can increase the risk of CVD along multiple pathways. The earliest sign of obesity-associated CVD is impaired NO-mediated relaxation, which leads to cardiovascular stiffness [5]. More notably, arterial stiffness—a feature of physiological vascular aging—is accelerated in various pathological conditions associated with increased cardiovascular risk and is a key risk factor for many cardiovascular diseases [6]. As a non-invasive and user-friendly method for estimating arterial stiffness, brachial-ankle pulse wave velocity (baPWV) is a valid and reproducible measure [7, 8].

BMI is a risk factor for a shorter healthy lifespan and increased overall CVD-related morbidity and mortality [9]. However, BMI is limited because it does not distinguish between fat depots, muscle, and skeletal weight [10]. Humans exhibit significant variability in body fat distribution for a given BMI [11], and fat distribution is a crucial determinant of CVD risk [5]. Relevant measures of fat distribution include percentage body fat (PBF), which is calculated by dividing total body fat by total body mass and multiplying by 100 [12]. As a marker of abdominal adiposity, waist-to-hip ratio (WHR) is an index of central obesity [13]. There is evidence that subcutaneous adipose tissue (SAT) and visceral adipose tissue (VAT) are both associated with metabolic risk factors and that VAT measurement provides a complete picture of metabolic risk associated with changes in fat distribution [14]. Another study also found that visceral and ectopic fat accumulation contributes significantly to cardiovascular and metabolic risk [11]. In summary, fat distribution is a more significant issue than overall body weight. However, measures of fat distribution vary in emphasis, and further studies are needed.

There is no doubt that fat distribution and arterial stiffness are closely related to age, and there is also evidence of sexual dimorphism in body fat distribution and cardiometabolic health [15, 16]. Few studies have investigated age and sex differences in fat distribution, especially in the Chinese population. Many studies have investigated the association between anthropometric indicators and arterial stiffness [17–22]. However, most of the existing studies were based on small samples and used indicators of fat distribution such as PBF, waist circumference (WC), hip circumference (HC), and WHR; only a few looked at the relationship between subcutaneous fat area (SFA), visceral fat area (VFA), and arterial stiffness. In this study, we used VFA, SFA, and anthropometric indices, and the number of study subjects was vast. The purpose of this study was to investigate differences in fat distribution and baPWV among Beijing adults aged 20–59 years by gender and age group, as well as the relationship between fat distribution and baPWV.

## Methods

### Participants

The study participants (N = 10,811) were individuals aged 20–59 years who were recruited to attend the physical examination in the physical health laboratory at the Workers Gymnasium (Dongcheng District, Beijing, China) between January 2015 and December 2018. All were working in Beijing and came from diverse backgrounds. Candidates with a history of coronary artery disease, cerebrovascular disease, peripheral vascular disease, severe liver or kidney disease, thyroid dysfunction, or malignant tumors were excluded from the study. All subjects signed an informed consent form, and the study complied with medical ethics standards.

### Anthropometric indices

A trained investigator measured WC and HC. WC was measured following exhalation at the midpoint between the lowest rib and iliac cres [23]. HC was measured at the point of maximum prominence. Both were measured to the closest 0.1 cm using a standard body tape. WHR was calculated by dividing WC by HC.

BMI and PBF were assessed using a multi-frequency bioelectrical impedance analyzer (JAWON X-scan Plus II, Seoul, South Korea). Before the test, subjects fasted for 8 h and urinated and defecated. They wore light clothing, removed their belongings, and avoided wearing metal objects. After 5 min of rest, the subject stepped barefoot onto an electrode at the designated position on the instrument. After the tester had entered information that included height, sex, and age, the subject gently grasped the handle electrode, with straight arms at an angle of 15 degrees to the torso. Subjects were asked to remain upright and stationary throughout the test.

SFA and VFA were measured using a DualScan® (HDS-2000, Omron, Kyoto, Japan) [24]. The DualScan® measures VFA and SFA by performing a dual bioelectrical impedance analysis (BIA) without radiation. Dual BIA instrument can be used to measure intra-abdominal fat area in obese patients, allows frequent measurement [25]. Before being measured, all participants fasted for at least eight hours and urinated and defecated. During measurement, participants were placed supine on a measuring mat, relaxing their whole body to ensure a calm state. A trained investigator then measured VFA and SFA in accordance with the manufacturer’s operating instructions.

### Measurement of baPWV

Each participant’s baPWV was measured using a BP-203 RPE III networked arteriosclerosis detection device [Omron Health Medical (China) Co., Ltd. (Dalian, China)], as detailed elsewhere [26]. Participants were prohibited from smoking, drinking, or consuming caffeinated beverages for at least three hours before measurement. After resting in a temperature-controlled room for 5 min, each participant lay on their back on the examination table and remained quiet during measurement. Trained investigators wrapped cuffs around the upper arm and ankle, leaving a finger gap with the limb. The lower edges of the arm cuffs were positioned 2–3 cm above the transverse striation of the cubital fossa; the lower edges of the ankle cuffs were positioned 1–2 cm above the superior aspect of the medial malleolus. A heart sound detector was then placed at the left edge of the sternum. Measurements were repeated for each observation, and the second was taken as the final result. The side with the higher baPWV on both sides was included in the analysis.

### Statistical analysis

All analyses were performed using SPSS 26.0 statistical software. For the initial descriptive analysis, continuous variables are reported as medians (quantiles). The Mann–Whitney U test was used to analyze differences between males and females, and the Kruskal–Wallis H test was used to analyze differences between age groups. Spearman’s correlation coefficient was then used to assess the correlation between age, BMI, PBF, WHR, SFA, VFA, and baPWV. Finally, a quantile regression was performed, using BaPWV as the dependent variable and age, BMI, PBF, WHR, SFA, and VFA as covariates. A two-sided *p* < 0.05 was considered significant.

## Results

### Body fat parameters and baPWV by sex and age

Table 1 lists participant characteristics by sex and age. Values of the fat parameters PBF, WHR, SFA, and VFA were significantly higher among older women when compared to those in the younger age group. BMI values for 50-year-old women did not differ significantly from those for 40-year-olds; in other groups, BMI values were significantly higher for the older age group than for the younger age group. WHR values were higher for older men than for younger age groups. While VFA values are also generally higher for older men, there was no significant difference in this regard between 50-year-old and 40-year-old men. BMI and SAT values were lower for 20-year-olds than for other groups, with no significant differences among those other groups. PBF values for male 20-year-olds were lower than for all other groups; these values were significantly higher for 50-year-olds than for 30-year-olds. In general, men’s PBF values are significantly lower than women’s, but their BMI, WHR, SFA, and VFA values are significantly higher. For both males and females, baPWV values were significantly higher in the older age group than in the younger age group. In addition, men’s baPWV values were significantly higher than those of women in the same age group.

**Table 1:** Characteristics of participants by sex and age

		Total	20–29 Years	30–39 Years	40–49 Years	50–59 Years
		(M = 3995, F = 6816)	(M = 764, F = 1383)	(M = 1331, F = 2295)	(M = 1040, F = 2247)	(M = 860, F = 891)
Age	Male	39 (31–48)	27 (25–28)	34 (32–37) ^a^	45 (42–47) ^ab^	54 (52–57) ^abc^
(years)	Female	38 (31–46) *	27 (25–28)	34 (32–37) ^a^	45 (42–47) ^ab^	52 (51–54) ^abc^ *
	Total	38 (31–47)	27 (25–28)	34 (32–37) ^a^	45 (42–47) ^ab^	53 (51–55) ^abc^
BMI	Male	25.4 (23.4–27.9)	24.5 (22.0–27.3)	25.6 (23.5–28.2) ^a^	25.7 (23.9–28.0) ^a^	25.6 (23.7–27.7) ^a^
(kg/m^2^)	Female	22.8 (20.8–25.2) *	21.3 (19.5–23.7) *	22.3 (20.4–24.8) ^a^ *	23.6 (21.8–25.7) ^ab^ *	23.9 (22.1–26.0) ^ab^ *
	Total	23.8 (21.5–26.4)	22.3 (20.1–25.3)	23.6 (21.1–26.4) ^a^	24.3 (22.3–26.7) ^ab^	24.7 (22.9–26.9) ^abc^
PBF	Male	23.0 (19.4–26.3)	21.0 (16.1–25.4)	23.0 (19.5–26.4) ^a^	23.4 (20.4–26.1) ^a^	23.7 (20.8–26.6) ^ab^
(%)	Female	27.1 (23.4–30.8) *	24.5 (20.6–28.6) *	26.2 (22.6–30.1) ^a^ *	28.3 (25.3–31.4) ^ab^ *	29.4 (26.4–32.4) ^abc^ *
	Total	25.5 (21.7–29.3)	23.3 (19.0–27.5)	25.1 (21.4–28.8) ^a^	26.7 (23.3–30.2) ^ab^	26.5 (22.9–30.0)^ab^
WHR	Male	0.90 (0.84–0.93)	0.83 (0.79–0.84)	0.87 (0.84–0.90) ^a^	0.92 (0.91–0.95) ^ab^	0.94 (0.92–0.97) ^abc^
	Female	0.79 (0.73–0.82) *	0.73 (0.71–0.79) *	0.77 (0.73–0.81) ^a^ *	0.80 (0.77–0.83) ^ab^ *	0.82 (0.80–0.84) ^abc^ *
	Total	0.81 (0.76–0.87)	0.77 (0.72–0.82)	0.80 (0.74–0.85) ^a^	0.83 (0.79–0.90) ^ab^	0.87 (0.82–0.94) ^abc^
SFA	Male	182.3 (141.9–226.5)	167.6 (120.0–232.5)	185.8 (144.5–233.0) ^a^	182.5 (147.7–223.1) ^a^	184.6 (150.0–221.2) ^a^
(cm^2^)	Female	160.35 (118.0–207.6) *	137.9 (95.2–189.8) *	153.6 (110.3–202.2) ^a^ *	167.5 (128.9–209.6) ^ab^ *	187.4 (149.0–226.6) ^abc^
	Total	169.2 (126.0–215.0)	147.8 (100.7–204.8)	165.9 (121.5–214.8) ^a^	173.3 (133.4–214.6) ^ab^	186.0 (149.5–223.7) ^abc^
VFA	Male	83.6 (59.4–113.1)	66.7 (44.4–95.9)	81.9 (57.8–108.7) ^a^	89.5 (66.1–117.9) ^ab^	94.6 (69.9–124.8) ^ab^
(cm^2^)	Female	50.1 (34.5–70.9) *	39.8 (28.1–54.9) *	45.0 (32.1–64.0) ^a^ *	56.5 (40.8–78.0) ^ab^ *	66.7 (48.6–88.3) ^abc^ *
	Total	60.6 (39.9–88.2)	46.5 (31.5–69.2)	55.7 (36.7–83.2) ^a^	65.5 (45.7–91.0) ^ab^	78.2 (56.2–106.8) ^abc^
baPWV	Male	1297 (1185–1428)	1187 (1097–1293)	1259 (1164–1369) ^a^	1339 (1228–1475) ^ab^	1400 (1288–1552) ^abc^
(cm/s)	Female	1152 (1057–1272) *	1060 (995–1136) *	1107 (1038–1195) ^a^ *	1226 (1132–1332) ^ab^ *	1301 (1188–1429) ^abc^ *
	Total	1205 (1092–1337)	1098 (1021–1196)	1158 (1067–1275) ^a^	1257 (1156–1381) ^ab^	1348 (1228–1492) ^abc^

Median values (25–75th percentile). M: Male; F: Female; BMI: body mass index; PBF: percentage body fat; WHR: waist-to-hip ratio; SFA: subcutaneous fat area; VFA: visceral fat area; baPWV: brachial-ankle pulse wave velocity. * *p* < 0.05, comparison between females and males. ^a^*p* < 0.05, comparison between groups and 20–29 age group. ^b^*p* < 0.05, comparison between groups and 30–39 age group. ^c^*p* < 0.05, comparison between groups and 40–49 age group.

### Correlations between variables

**Fig. 1:**
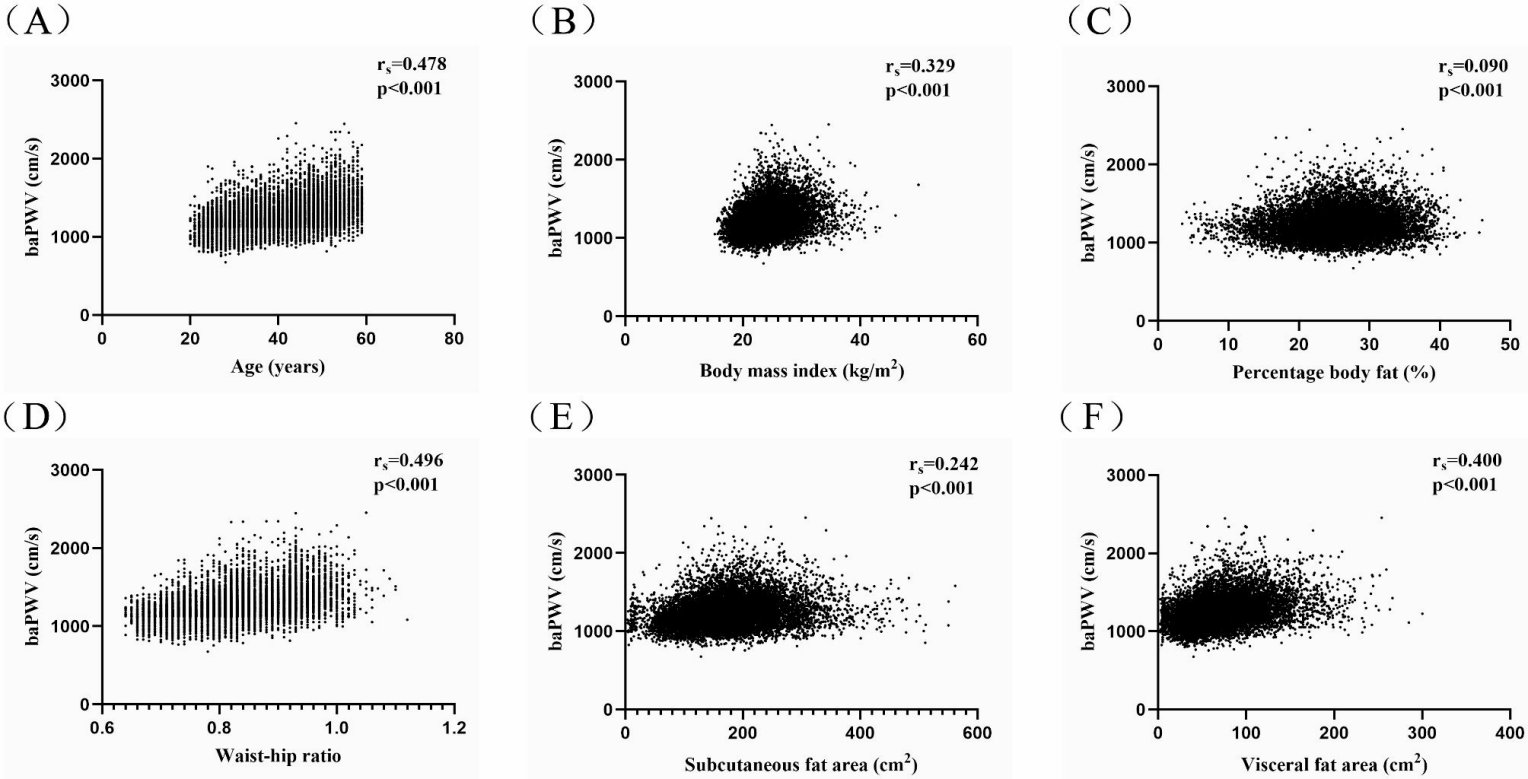
Scatter plots showing correlations of brachial-ankle pulse wave velocity (baPWV) with (**A**) age, (**B**) body mass index, (**C**) percentage body fat, (**D**) waist-to-hip ratio, (**E**) subcutaneous fat area, and (**F**) visceral fat area

Table 2 shows the correlations between age, anthropometric indicators (BMI, PBF, WHR, SFA, VFA), and baPWV. The results indicate that while age is significantly and positively correlated with anthropometric indicators and baPWV, it is more strongly correlated with the latter (rs = 0.478, *p* < 0.001). In addition, all anthropometric indicators are positively correlated to varying degrees. Anthropometric indicators are all significantly and positively correlated with baPWV; WHR (rs = 0.496, *p* < 0.001) and VFA (rs = 0.400, *p* < 0.001) are more strongly correlated with baPWV, followed by BMI (rs = 0.329, *p* < 0.001), SFA (rs = 0.242, *p* < 0.001), and PBF (rs = 0.090, *p* < 0.001). Figure 1 shows each indicator’s correlation with baPWV.

**Table 2:** Correlations: Anthropometric indices and baPWV

Variable	BMI	PBF	WHR	SFA	VFA	baPWV
Total						
Age	0.21 **	0.20 **	0.44 **	0.16 **	0.28 **	0.48 **
BMI		0.65 **	0.79 **	0.78 **	0.70 **	0.33 **
PBF			0.35 **	0.66 **	0.36 **	0.09 **
WHR				0.61 **	0.69 **	0.50 **
SFA					0.58 **	0.24 **
VFA						0.40 **
Female						
Age	0.28 **	0.30 **	0.47 **	0.22 **	0.32 **	0.54 **
BMI		0.93 **	0.88 **	0.77 **	0.62 **	0.27 **
PBF			0.94 **	0.79 **	0.59 **	0.29 **
WHR				0.74 **	0.59 **	0.37 **
SFA					0.50 **	0.23 **
VFA						0.31 **
Male						
Age	0.08 **	0.14 **	0.71 **	0.05 **	0.23 **	0.42 **
BMI		0.84 **	0.58 **	0.82 **	0.70 **	0.15 **
PBF			0.70 **	0.78 **	0.67 **	0.20 **
WHR				0.51 **	0.58 **	0.40 **
SFA					0.69 **	0.14 **
VFA						0.25 **

BMI: body mass index; PBF: percentage body fat; WHR: waist-to-hip ratio; SFA: subcutaneous fat area; VFA: visceral fat area; baPWV: brachial-ankle pulse wave velocity. ** *p* < 0.01.

### Quantile regression

**Fig. 2:**
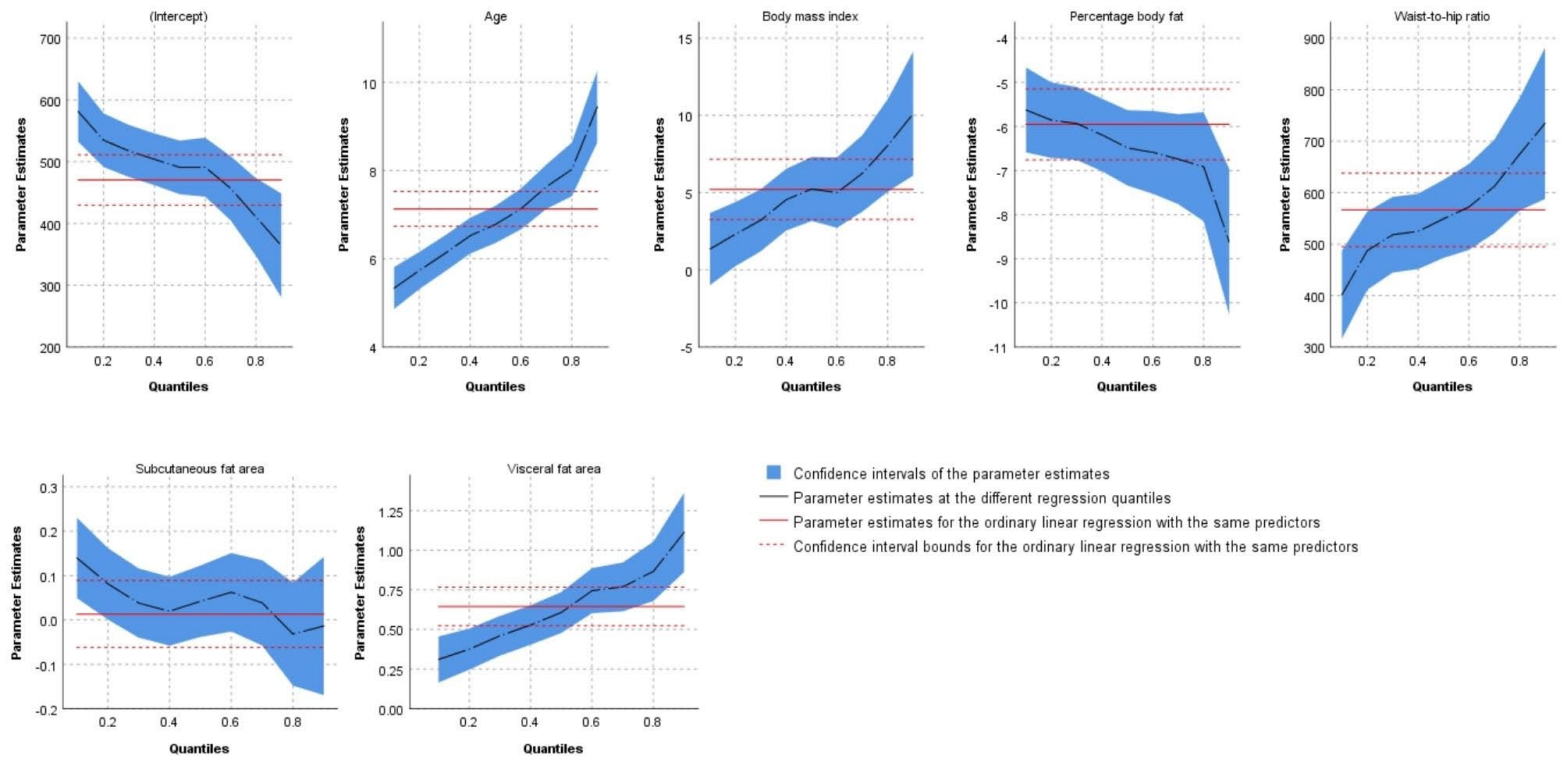
Quantile regression plots of predictor effect on quantiles of brachial-ankle pulse wave velocity

Table 3 shows coefficients of quantile regression, with BaPWV as the dependent variable, and age, BMI, PBF, WHR, SFA, and VFA as covariates. Quantile regression was performed to assess the covariates’ association with baPWV for different quantiles. The results indicate that the coefficients of age, BMI, PBF, WHR, and VFA were significant for all quantiles of baPWV and that the effects of age, PBF, WHR, and VFA on baPWV were greater for the higher quantiles. Although BMI’s coefficient decreased in the 60th quantile, the overall trend increased. SFA’s coefficients were significant for lower quantiles (q ≤ 0.2). Details of covariate effect trends are shown in Fig. 2.

**Table 3:** Results of quantile regression analysis for predictors of baPWV.

Variable	Quantiles								
	q = 0.1	q = 0.2	q = 0.3	q = 0.4	q = 0.5	q = 0.6	q = 0.7	q = 0.8	q = 0.9
Age	5.335 **	5.738 **	6.121 **	6.526 **	6.782 **	7.133 **	7.635 **	8.026 **	9.446 **
BMI	1.349	2.311 *	3.213 **	4.541 **	5.235 **	5.002 **	6.250 **	8.054 **	10.118 **
PBF	−5.628 **	−5.859 **	−5.934 **	−6.195 **	−6.488 **	−6.588 **	−6.743 **	−6.915 **	−8.616 **
WHR	401.715 **	487.365 **	518.156 **	524.758 **	549.055 **	572.075 **	611.960 **	674.735 **	734.596 **
SFA	0.140 **	0.082 *	0.038	0.020	0.042	0.062	0.039	−0.032	−0.013
VFA	0.311 **	0.376 **	0.460 **	0.528 **	0.608 **	0.745 **	0.769 **	0.867 **	1.112 **
Intercept	581.335 **	534.742 **	517.295 **	503.961 **	490.788 **	491.058 **	457.111 **	410.646 **	364.366 **

BMI: body mass index; PBF: percentage body fat; WHR: waist-to-hip ratio; SFA: subcutaneous fat area; VFA: visceral fat area; baPWV: brachial-ankle pulse wave velocity. * *p* < 0.05, ** *p* < 0.01.

## Discussion

Our findings indicate that fat accumulates with age and that men tend to accumulate more abdominal fat than women in the same age group. Similarly, baPWV increases with age and is significantly higher among men than among women in the same age group. We also found that baPWV is positively correlated with age, BMI, PBF, WHR, SFA, and VFA. In addition, the correlation between baPWV and age, WHR, and VFA is stronger than the correlation between BMI, PBF, and SFA. Finally, the effects on baPWV of age, BMI, PBF, WHR, and VFA are greater at higher quantiles of baPWV.

### Age and sex differences in fat distribution

The higher fat parameter values observed in older age groups—and especially among women—suggest that fat accumulation increases with age. This finding aligns with an earlier cross-sectional study of adults in southern China, which found that whole-body fat mass, PBF, android fat mass, and android/gynoid fat mass ratio increase with age [27]. These age-related changes may be linked to resting metabolic rate (RMR); as we age, decreases in individual organ/tissue mass and tissue-specific organ metabolic rate contribute to a decrease in RMR, favoring an increase in fat mass [28]. Physical activity and exercise also play an important role in the regulation of human adipose tissue physiology [29]. For example, there is evidence that physical activity level (based on total energy expenditure as a multiple of resting energy expenditure) increases gradually from early age to adulthood and decreases again in old age [30]. On comparing how a number of fat parameters change with age, we identified significant differences in WHR across different groups (both male and female). Our analysis also shows that age is more strongly correlated with WHR than with other fat parameters—that is, WHR increases significantly with age.

As well as age differences, we observed sex differences in fat distribution within the study population. While men’s PBF values were lower than those of women in the same age group, men’s BMI, WHR, and VFA values were higher than those of women in the same age group. These findings align with an earlier study of gender differences in body composition, which reported that women’s PBF values were higher than men’s while VAT values were higher for men [31]. In addition, we observed a nonsignificant age-related change in men’s SFA values across three age groups (30–59 years), as well as significant variability among women (all groups). These results suggest that men are more likely to accumulate visceral fat, leading to abdominal fat accrual. This gender difference in fat distribution may relate to sex hormones and sex hormone receptors; estrogen differentially augments sympathetic tone to the adipose tissue depots, favoring the accumulation of subcutaneous fat in women and the deposition of visceral fat in men [32]. Moreover, female subcutaneous adipose tissue contains more estrogen receptors than androgen receptors. In visceral adipose tissue, androgen receptor expression in males increases relative to estrogen receptors [15].

### Differences in baPWV by age and sex

In this study, we found that baPWV was significantly higher in the older age group (both male and female). We also found that baPWV was significantly and positively correlated with age and that baPWV in the higher quantiles was more affected by age. Our results are consistent with the findings of a large-scale cross-sectional study which found that baPWV increases with age, implying that arterial stiffness increases with age [33]. The association between age and arterial stiffness can be understood in terms of both structure and function. Structurally, the walls of large conduit arteries (especially the aorta) thicken and lose elasticity as we age, leading to an increase in pulse wave velocit [34]. Functionally, aging is associated with increased incidence of metabolic syndrome, increased inflammation, and neurohormonal dysfunction, all of which promote arterial stiffening and accelerate vascular aging [34]. One earlier study also found that oxidative stress may be independently associated with arterial stiffness in subjects aged over 45, suggesting that it is vital to reduce oxidative stress in order to prevent age-related structural and functional arterial changes and subsequent disease [35]. Comparing our findings with previous studies, we found that baPWV among men in Beijing is higher than among Japanese and American males of the same age. Among the women in our study, baPWV was higher than in Japan and closer to values in the United State [36]. We surmise that these differences relate mainly to lifestyle differences, but in the absence of original data, this can only be confirmed by experiments with larger samples.

Regarding sex differences, we found that baPWV was significantly higher in men than in women in the same age group. Another study reported similar results, showing that men aged 18–50 had much higher baPWV values than women in the same age group [33]. Gender differences in arterial stiffness are also inextricably linked to sex hormones. According to some previous studies, the female sex hormone estrogen and associated receptors expressed in the vasculature and heart have a cardioprotective effect by increasing angiogenesis and vasodilation and reducing ROS, oxidative stress, and fibrosis [37–39]. There is also evidence that normal physiological levels of testosterone, the primary male sex hormone, may protect the male cardiovascular system while testosterone deficiency is associated with an unfavorable metabolic profile and more cardiovascular events [40].

It is important to note that androgens differ in their effects on males and females. In women, the key factor is the estradiol–testosterone ratio, while men are affected by the aromatization of androgens into estrogens [41]. As the female cardiovascular system is strongly influenced by menopause, and our participants were aged 20–59 years, we reviewed the relevant literature. One study suggested that menopause is an independent factor that augments age-related increase in arterial stiffness in the early postmenopause [42]. Another study examining the trajectories of age-related arterial stiffness in Chinese men and women found that it is associated with higher PWV in men as compared to premenopausal women and with higher PWV in postmenopausal women as compared to men [43]. This difference may relate to factors such as iron storage, oxidative stress, and estrogen deficiency [42, 44, 45].

### Relationship between fat distribution and baPWV

Our findings indicate that baPWV is positively correlated with BMI, PBF, WHR, VFA, and SFA. The possible reason is that as well as directly altering the structure and function of the cardiovascular system, obesity can lead to insulin resistance, hyperglycemia, hypertension, and other cardiovascular disease risk factors, indirectly increasing CVD morbidity and mortality [46]. More importantly, baPWV was more strongly correlated with WHR and VFA than with BMI, PBF, and SFA. This suggests that abdominal obesity is more closely associated with arterial stiffness than overall obesity, which is generally consistent with previous studies [22, 47]. Among a number of mechanisms that might explain this, the most likely candidate is inflammatory action. Adipose tissue is a rich source of inflammatory cytokines, and there is evidence that visceral fat secretes higher levels of pro-inflammatory factors than subcutaneous fat [48]. Inflammation plays an important role in the development of arterial stiffness by affecting the function of arterial endothelial or smooth muscle cells and the structure of the arterial wall [49]. One concern is that the expansion of adipose tissue can lead to hyperleptinemia and leptin resistance [50]. An earlier study reported that leptin-mediated aldosterone secretion promotes endothelial dysfunction and the expression of pro-fibrotic markers in the heart, leading to cardiovascular disease [51]. In addition, chronic pro-inflammation caused by obesity plays a key role in the development of insulin resistance [52]. A Chinese population-based study found that levels of insulin resistance were proportional to levels of arterial stiffnes [53].

Interestingly, we observed that baPWV is more strongly correlated with WHR than with VFA. A meta-regression analysis of prospective studies also indicated that WHR and WC are significantly associated with risk of incident CVD event [54]. This suggests that WHR should be included in CVD risk assessments as a simple measure of abdominal obesity. Using quantile regression, we also explored the effects of adiposity parameters on different baPWV populations; our results show that populations with higher baPWV—that is, those with pre-existing arterial stiffness—are more affected by adiposity parameters. This may be because people with higher baPWV already have other diseases and are more susceptible to risk factors. In summary, maintaining cardiovascular health depends on reducing fat accumulation, especially visceral fat.

### Strengths and limitations

The main strength of the present study is its large sample size. In addition, we used quantile regression to analyze the effect of fat distribution on different levels of baPWV, which enabled us to explore the relationship between the independent and dependent variables more comprehensively and in greater detail, and ensured that the results of our analysis were more robust. The study also has some limitations. First, the cross-sectional design made it impossible to infer causal relationships. Second, the study was confined to Beijing, and further research is needed to generalize the results to other places. Third, the cardiovascular system is affected by menopause, but our study population ranged in age from 20 to 59 years. Finally, we did not consider the participants’ lifestyles, such as alcohol use, physical activity, eating habits, or sleep deprivation.

## Conclusions

Our findings indicate that fat accumulates with age and that men tend to accumulate more abdominal fat than women in the same age group. We also found that baPWV increases with age and is significantly higher among men than among women in the same age group. In addition, the correlations between WHR, VFA, and baPWV were stronger than those between baPWV and BMI, SFA, and PBF, indicating that abdominal obesity is more closely linked to arterial stiffness than overall obesity. Finally, the finding that people with higher baPWV are more affected by obesity parameters suggests there is a greater need to control obesity-related risk factors in people with arterial stiffness.

In the current study, we did not consider the participants’ lifestyles, which require in-depth investigation in future studies. In addition, the prevalence of cardiovascular disease comorbid with diabetes is severe and should be paid attention to in future studies. Finally, future research should focus more on the elderly population, which is essential to understand increases in baPWV. Our findings have some policy implications for chronic disease prevention and control. First of all, abdominal obesity is more harmful than overall obesity and should focus on the health of abdominal obese people. Secondly, early screening and early intervention for chronic diseases are essential. Finally, the government should increase publicity to make people aware of the hazards of obesity.

### Electronic supplementary material

Below is the link to the electronic supplementary material.

Supplementary Material 1: Table S1: STROBE Statement—Checklist of items that should be included in reports of cross-sectional studies.

## Data Availability

The datasets used and/or analysed during the current study are available from the corresponding author on reasonable request.
